# Exogenous Melatonin Enhances the Low Phosphorus Tolerance of Barley Roots of Different Genotypes

**DOI:** 10.3390/cells12101397

**Published:** 2023-05-16

**Authors:** Zengke Ma, Ke Yang, Juncheng Wang, Jingwei Ma, Lirong Yao, Erjing Si, Baochun Li, Xiaole Ma, Xunwu Shang, Yaxiong Meng, Huajun Wang

**Affiliations:** 1 State Key Lab of Aridland Crop Science/Gansu Key Lab of Crop Improvement and Germplasm Enhancement, Lanzhou 730070, China; zengkema@sina.com (Z.M.); yangk@gsau.edu.cn (K.Y.);; 2 Department of Crop Genetics and Breeding, College of Agronomy, Gansu Agricultural University, Lanzhou 730070, China; 3 Department of Botany, College of Life Sciences and Technology, Gansu Agricultural University, Lanzhou 730070, China

**Keywords:** barley, melatonin, metabolomic, low phosphorus stress, antioxidant, P remobilization

## Abstract

Melatonin (N-acetyl-5-methoxytryptamine) plays an important role in plant growth and development, and in the response to various abiotic stresses. However, its role in the responses of barley to low phosphorus (LP) stress remains largely unknown. In the present study, we investigated the root phenotypes and metabolic patterns of LP-tolerant (GN121) and LP-sensitive (GN42) barley genotypes under normal P, LP, and LP with exogenous melatonin (30 μM) conditions. We found that melatonin improved barley tolerance to LP mainly by increasing root length. Untargeted metabolomic analysis showed that metabolites such as carboxylic acids and derivatives, fatty acyls, organooxygen compounds, benzene and substituted derivatives were involved in the LP stress response of barley roots, while melatonin mainly regulated indoles and derivatives, organooxygen compounds, and glycerophospholipids to alleviate LP stress. Interestingly, exogenous melatonin showed different metabolic patterns in different genotypes of barley in response to LP stress. In GN42, exogenous melatonin mainly promotes hormone-mediated root growth and increases antioxidant capacity to cope with LP damage, while in GN121, it mainly promotes the P remobilization to supplement phosphate in roots. Our study revealed the protective mechanisms of exogenous MT in alleviating LP stress of different genotypes of barley, which can be used in the production of phosphorus-deficient crops.

## 1. Introduction

Phosphorus (P) is an essential macronutrient for plant growth and development, and is involved in many physiological processes [1]. It is particularly important to regulate the synthetic and metabolic pathways of some important compounds, such as membrane lipids, nucleic acids, ATP, and phosphorylation metabolites [2]. Although soil is, overall, rich in P, most of it cannot be absorbed by plants due to its low diffusion rate and instability of forms [3]. Pi deficiency is one of the main limiting factors affecting crop yield worldwide, with high-Pi fertilizer used on more than 30% of the world’s arable land for optimum crop production [4]. However, excessive application of P fertilizer causes serious environmental problems and affects the sustainability of phosphorus resources [5]. Plants take up P mainly from the soil in the form of inorganic phosphate (Pi) via their roots [6], but there are large differences in the distribution of Pi between plant cells (mM) and soil solution (µM) [7]. Although plants have evolved a variety of morphological and physiological strategies to respond to phosphorus deficiency stress [8], such as root remodeling, increased uptake of phosphorus in the soil, and coordination of phosphorus transport and redistribution in plants, these strategies are limited under severe phosphorus deficiency [9]. In addition to chemical fertilizers, adding biological regulators to control various environmental stresses in crop production is an important means of achieving sustainable agricultural development.

Melatonin is a low-molecular-weight indoleamine substance that was first detected in the pineal gland of cows [10]. However, it was not discovered and isolated from vascular plants until 1995 [11,12]. Recently, the biosynthetic and metabolic pathways of melatonin in plants have been well characterized. Its synthesis begins with tryptophan, followed by four sequential enzyme reactions involving at least six enzymes [8,13,14]. Thereafter, melatonin is converted into 2-hydroxymelatonin, cyclic-3-hydroxymelatonin, or AFMK by enzymatic action or interaction with ROS [15,16]. The recent discovery of the first plant melatonin receptor (CAND2/PMTR1) in *Arabidopsis thaliana* has led researchers to consider melatonin as a new plant hormone to further identify its functions and signal transduction pathways [17]. To date, evidence suggests that melatonin plays a regulatory role in plant growth, seed germination, root and flower development, photosynthesis, and circadian rhythms [18]. Importantly, melatonin is considered to be the first line of defense against different biotic and abiotic stresses such as drought [19], salinity [20], cold and high temperatures [21,22], pathogens [23], and nutrient deficiency [24,25]. For example, in terms of defense against nutritional stress, Chen et al. [26] reported that the application of exogenous melatonin enhanced the tolerance of bermudagrass to low-potassium stress. In addition, a recent study in *Malus hupehensis* also revealed that exogenous melatonin enhances tolerance to low nitrogen by regulating root architecture, N metabolism and related gene expression [27]. Melatonin protects plants by maintaining the homeostasis of reactive oxygen species and activating various antioxidant enzymes, forming antioxidant cascade reactions [28,29]; these effects are based on its auxin-like role and antioxidant properties [30]. Based on these findings, the direct application of exogenous melatonin or regulation of endogenous melatonin metabolism was considered a feasible and cost-effective method of promoting plant growth or improving abiotic stress resistance [31].

In the long term, high-fertility species result in crop varieties with good tolerance to nutrient limitations often being eliminated [32]. Barley is one of the earliest domesticated crops and currently ranks fourth in global grain production [33]. Fortunately, barley still shows a wide adaptation to extreme environments compared with other cereal crops, especially its low nutrient requirements. In fact, this led to this ancient cereal being considered an advantageous species for studying the mechanisms of nutrient deficiency tolerance [34]. Over the last two decades, metabolomics has rapidly emerged as a key tool for gene function annotation, which has greatly promoted the comprehensive understanding of cell responses to changes in biological conditions. Previous studies confirmed that melatonin regulates different metabolic pathways in plants, such as carbohydrate, lipid, nitrogen, and phosphorus cycles [35,36]. Additionally, several secondary metabolites, such as simple phenols, flavonoids, anthocyanins, linoleic acid, and terpenoids, were found to be increased after the application of exogenous melatonin under stress conditions [37,38]. To date, many metabolites have been characterized as being involved in the Pi starvation response of plants [39,40,41]. Xu et al. [25] reported that melatonin may be involved in citric acid cycle, amino acid, polyamine, linoleic acid and organic acid metabolism of tomato, thus alleviating the damage of LP stress on tomato growth. However, melatonin levels in plants vary widely among species, varieties, and circadian rhythms, among other things. To our knowledge, there has been little research on the beneficial role of melatonin in barley seedlings and different tolerance genotypes under LP stress.

In this study, we aimed (1) to identify the changes of root structure characteristics, antioxidant system and hormone content of exogenous melatonin-treated barley seedlings of different tolerance genotypes and (2) to identify the key metabolites that alleviated LP stress. Our study provides novel insights into the molecular mechanism by which melatonin enhances LP tolerance, which may also have important applications in alleviating nutritional stress in barley as well as other crops.

## 2. Materials and Methods

### 2.1. Plant Material and Experimental Design

The cultivated barley GN121 (LP-tolerant) and GN42 (LP-sensitive), which were obtained from the College of Agronomy, Gansu Agricultural University, were used as experimental materials [42]. Seeds were surface-disinfested with 3% NaClO solution for 10 min, then rinsed with distilled water, followed by germination in a transparent plastic germinator (18 × 13 × 11 cm) with two layers of wet filter paper. After 7 days, seedlings of uniform growth status were transplanted into a modified Hoagland hydroponic nutrient solution and subjected to the following three treatments: normal-phosphorus nutrient solution (CK, 0.397 mM KH_2_PO_4_), low-phosphorus nutrient solution (LP, 0.0397 mM KH_2_PO_4_), and low-phosphorus stress and exogenous melatonin treatment (LP+MT, 0.0397 mM KH_2_PO_4_ + 30 μM melatonin). The concentration of exogenous melatonin at 30 μM was determined based on our preliminary experimental results (data not shown), and which is an active and suitable dose. The phosphorus concentration, composition of other nutrient elements and pH in hydroponic solutions were the same as described by Ren et al. [43]. The seedlings were grown in a phytotron with a 16 h light/8 h dark photoperiod, radiation intensity of approximately 300 μmol m^−2^ s^−1^, temperature of 20 ± 5 °C, and relative humidity of 50–70%. The nutrient solution was continuously aerated with pumps and renewed every 3 days. After the 15 treatment days, roots were separated and rinsed with distilled water. Each treatment had nine biological replicates and four individuals from each replicate were pooled as a composite sample. All barley roots were quickly frozen in liquid nitrogen after collection for physiological and metabolomic analyses.

### 2.2. Root Morphology and Phosphorus Concentration

A root automatic scanner (1680; Epson, Long Beach, CA, USA) was used to scan the separated roots at 300 dpi. The images were analyzed using software WinRHIZO Pro (Regent Instruments Inc., Quebec, ON, Canada) to quantify root system architecture (RSA) traits, including total root length (TRL), root surface area (ROSA), and root volume (RV). The root tissues were powdered and digested, and phosphorus concentrations were determined by the method described by Chapman and Pratt [44].

### 2.3. Quantification of Melatonin by LC-MS

The extraction and analysis of endogenous plant hormones were determined according to the method reported by Pan et al. [45] with some modifications. Around 0.2 g tissue was ground in liquid nitrogen and the obtained powder was homogenized in 5 mL extraction solvent (2-propanol/H_2_O/concentrated HCl, 2:1:0.002, *v*/*v*/*v*) incubated for 30 min at 4 °C in darkness, and then mixed with methylene chloride (3 mL), incubated for another 30 min and centrifuged at 12,000 rpm for 5 min at 4 °C in darkness. The pooled supernatants (2 mL) were evaporated to dryness with a vacuum freezing concentrator (BioCool, Beijing, China), and then extract was dissolved in 80% methanol (1 mL). All samples were subjected to low light conditions and five replicates were performed for each treatment.

Five reference standards, i.e., melatonin (MT, SM8590), indoleacetic acid (IAA, S18340), abscisic acid (ABA, SA8750), gibberellic acid (GA_3_, SG8920), trans-zeatin (tZ, SZ8090), were purchased from Solarbio (Beijing, China), and dissolved in 80% methanol. All standard and extracted samples were run using an HPLC Infinity 1260 Quaternary LC system (Agilent Technologies, Waldbronn, Germany) and the chromatographic column was ORBAX SB-C18 (4.6 × 250 mm, 5 μm, Agilent, Santa Clara, CA, USA). The samples were injected and eluted by C (methanol) and D (0.1% phosphoric acid in water) of the quaternion gradient, with an injected quantity of 5 μL. The back pressure was less than 400 bar, column temperature was 30 °C, and flow rate was 1 mL/min. The 12 min linear gradient elution was as follows: 0 min, 10% eluent C, 90% eluent D; 1 min, 45% eluent C, 55% eluent D; 3 min, 55% eluent C, 45% eluent D; 5 min, 65% eluent C, 35% eluent D; 8 min, 75% eluent C, 25% eluent D; 10 min, 90% eluent C, 10% eluent D. At the end of the gradient, the column was equilibrated to initial conditions for 2 min. Plant hormones were identified by comparing the retention time and spectra of the peaks in standards. Data processing and quantification of hormones was performed using OpenLab CDS 2.5 software (Agilent, Santa Clara, CA, USA).

### 2.4. Determination of ROS Accumulation and Antioxidant Enzyme Activities

The oxygen free radical (O^2−^), hydrogen peroxide (H_2_O_2_), malondialdehyde (MDA), superoxide dismutase (SOD), peroxidase (POD) and catalase (CAT) kits were purchased from Solarbio (Beijing, China). Extraction and determination were performed in accordance with the manufacturer’s instructions. Briefly, each sample of fresh roots (around 0.1 g) was supplemented with 1 mL of extraction solution and ground into a homogenate in an ice bath. After centrifugation at 8000× *g* for 20 min at 4 °C, the supernatant was collected and stored at 4 °C for activity analysis. The histochemical analysis of O^2−^ and H_2_O_2_ was performed using nitroblue tetrazolium (NBT) and 3,3-diaminobenzidine (DAB), respectively [46,47].

### 2.5. Metabolite Extraction and LC-MS/MS Analysis

Metabolite extraction was performed according to the method described by Wang et al. [48]. Briefly, approximately 80 mg of barley root was ground with liquid nitrogen and the obtained powder was homogenized in 1 mL of methanol/acetonitrile/H_2_O (2:2:1, *v*/*v*/*v*), vortexed and centrifuged at 14,000× *g* for 15 min at 4 °C. The supernatant was collected in LC-MS vials transferred for UHPLC-QE orbitrap/MS analysis. The supernatant of each sample was mixed equally to make quality control (QC) sample.

The extracted compounds were analyzed using a UHPLC system (1290 Infinity LC; Agilent Technologies, Waldbronn, Germany) coupled with a TripleTOF 6600 (Ab Sciex, Framingham, MA, USA). For HILIC separation, samples were injected onto an ACQUITY UPLC BEH column (2.1 × 100 mm, 1.7 µm; Waters, Ireland) at 25 °C, and using a 21.2 min linear gradient at 0.3 mL/min flow rate and 2 μL injection volume. The mobile phase composition and gradient elution procedures are described by Liu et al. [49]. ESI source conditions were set as follows: curtain gas (CUR) as 30, ion source gas1 (GS1) as 60, and ion source gas2 (GS2); source gas temperature as 600 °C; ISVF as 5500 V. The MS/MS acquisition was performed in positive (PI) and negative ion (NI) modes, the mass range was set at 25–1000 m/z, and the accumulation time for product ion scan was set at 0.05 s/spectrum. The second order spectrum was acquired in information-dependent acquisition (IDA) and high-sensitivity modes. Parameters were set as follows: declustering potential (DP) as ±60 V; collision energy as 35 V ± 15 eV; maximum candidate ions to monitor per cycle as 10; excluding isotopes within 4 Da.

### 2.6. Qualitative and Quantitative Analyses of Metabolites

The metabolite database search was described by Liu et al. [50]. Briefly, the Compound Discoverer 3.1 (CD3.1, Thermo Fisher, Waltham, MA, USA) was used for peak alignment, peak picking and quantification of the original data. The peaks obtained are then searched in the mzCloud (https://www.mzcloud.org/, accessed on 19 November 2022), mzVault and MassList database to identify metabolites. For preliminary visualization of the differential metabolites between different treatments, principal component analysis (PCA), partial least-squares discriminant analysis (PLS-DA), and orthogonal projection to latent structures-discriminant analysis (OPLS-DA) were performed on all samples using corresponding R package models. Those with a *p* value in the *t*-test of <0.05 and VIP ≥ 1 were identified as differential metabolites between the two groups. The z-score was used to normalize the abundance of differential metabolites in the same group. KEGG pathways were used for annotation and enrichment analysis (FDR ≤ 0.05).

### 2.7. Statistical Analysis

Means, standard errors, and the significance of differences were calculated using SPSS 22.0 software, followed by Duncan’s multiple-range tests (*p* < 0.05). Three and six biological repetitions were performed in the determination of physiological indexes and metabolomics, respectively. Software Origin Pro 8.0 and Adobe Photoshop CS6 are used to draw the figures.

## 3. Results

### 3.1. Exogenous Melatonin Improved the Primary Root Growth under LP Stress

**Figure 1:**
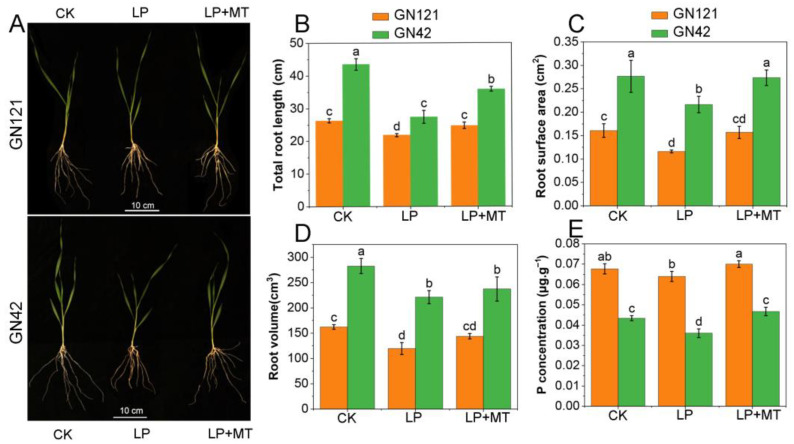
Exogenous melatonin promoted the tolerance of barley to LP stress. (**A**) The barley seedlings of GN121 and GN42 genotypes in 7 days were transferred to CK, P, and MT for 15 days of hydroponic culture. The root morphology indexes of the two barley genotypes included (**B**) TRL, (**C**) RSA, (**D**) RV, and (**E**) root P concentration. GN121, LP-tolerant barley; GN42, LP-sensitive barley; CK, normal Pi; LP, low Pi; LP+MT, low Pi + 30 μM melatonin. Results are mean ± SE (*n* = 3). Different letters above bars indicate statistically significant differences at *p* ≤ 0.05, based on Duncan’s multiple-range test.

To illustrate the effects of melatonin on barley root system architecture under LP stress, a series of treatments were performed for 15 days (Figure 1). A significant growth inhibitory effect was observed when the different genotypes of barley seedlings were treated with LP. Notably, pretreatment with 30 μM melatonin significantly reduced the inhibitory effects of LP stress on root growth. In detail, for GN121, compared with CK treatment, TRL, ROSA, and RV under LP treatment decreased by 17.0%, 28.0%, and 26.2%, while under LP+MT treatment they decreased by 5.9%, 2.5%, and 11.2%, respectively. In GN42, compared with CK treatment, TRL, ROSA, and RV under LP treatment decreased by 37.1%, 21.7%, and 21.8%, while under LP+MT treatment they decreased by 17.6%, 1.1%, and 16.0%, respectively. In addition, we noticed that melatonin promoted the P concentration under LP stress. These results revealed that exogenous melatonin may respond to LP stress by reducing root damage and P uptake in barley.

### 3.2. Exogenous Melatonin Regulates Changes in Endogenous Hormone Levels

**Figure 2:**
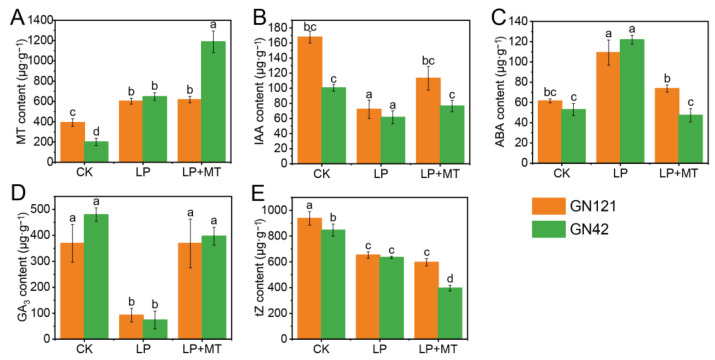
The contents of endogenous hormone in barley roots of GN121 and GN42 under CK, LP and LP+MT treatments. (**A**) MT (**B**) IAA (**C**) ABA (**D**) GA3 (**E**) tZ contents in roots under CK, LP, and LP+MT treatments. Results are the mean ± SE (*n* = 3). Different letters above bars indicate statistically significant differences at *p* ≤ 0.05, based on Duncan’s multiple-range test.

Compared with the CK treatment, LP stress significantly increased the contents of endogenous MT and ABA levels, and decreased the contents of IAA, GA3 and tZ in the roots of both barley genotypes (Figure 2). Notably, there was no significant difference in the contents of five endogenous hormones in the roots between the two genotypes under LP treatment, but the contents of endogenous MT, IAA and tZ under CK treatment were higher in GN121 than in GN42. Under LP treatment, exogenous melatonin did have a significant effect on endogenous MT, IAA, ABA, GA3 and tZ levels in GN42 but not MT, and tZ levels in GN121.

### 3.3. Exogenous Melatonin Alleviated ROS Accumulation against LP-Induced Oxidative Stress

**Figure 3:**
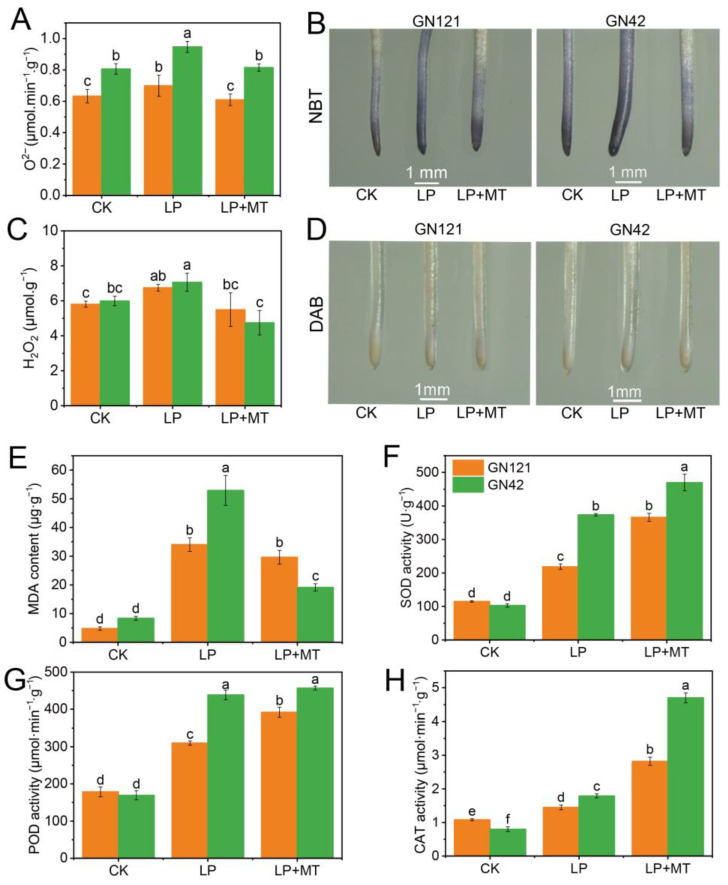
Accumulation of ROS and MDA and activities of antioxidant enzymes in barley roots of GN121 and GN42 under CK, LP and LP+MT treatments. (**A**) O^2−^ and (**C**) H_2_O_2_ (**E**) MDA contents in roots under CK, LP, and LP+MT treatments. (**B**) O^2−^ and (**D**) H_2_O_2_ accumulation was detected by nitroblue tetrazolium (NBT) (dark blue) and diaminobenzidine (DAB) (brown) staining. (**F**) SOD (**G**) POD (**H**) CAT activities in roots under CK, LP, and LP+MT treatments. Results are mean ± SE (*n* = 3). Different letters above bars indicate statistically significant differences at *p* ≤ 0.05, based on Duncan’s multiple-range test.

In order to evaluate the regulation effect of melatonin on LP-induced oxidative stress, the contents of O^2−^, H_2_O_2_ and MDA and the activity of antioxidant enzymes were examined (Figure 3A,C,E). Compared with CK treatment, LP treatment significantly induced the accumulation of O^2−^, H_2_O_2_ and MDA in barley roots, especially in GN42, which indicated that root cells suffered ROS damage and lipid peroxidation. However, exogenous melatonin promoted ROS scavenging and reduced lipid peroxidation levels. Compared with LP treatment, the contents of O^2−^, H_2_O_2_ and MDA under LP+MT treatment were decreased by 12.9%, 18.6% and 12.9% in GN121, respectively, and these decreased by 13.9%, 32.9% and 63.9% in GN42, respectively. As shown in Figure 3B,D, NBT and DAB histochemical staining showed the accumulation of O^2−^ and H_2_O_2_ under LP treatment and the scavenging under melatonin treatment, respectively. Apparently, the alleviation of melatonin to ROS accumulation is associated with activation of the antioxidant enzymes. The activity of SOD, POD and CAT in both barleys was significantly increased by exogenous melatonin treatment (Figure 3F–H). Compared with LP treatment, the activity of SOD, POD and CAT under LP+MT treatment are increased by 67.4%, 26.5% and 94.5% in GN121, respectively, and that decreased by 25.8%, 4.2% and 162.6% in GN42, respectively. It is worth noting that compared with GN21, GN42 maintained higher antioxidant enzyme activity under LP and LP+MT treatment, which may be related to its exposure to more severe oxidative stress.

### 3.4. Data Quality Analysis

To verify the accuracy of metabolomics, we extracted chromatograms of nine representative metabolites (Figure S1). To evaluate the stability of the analytical method and detection platform, PCA was used to reduce dimensionality and to visualize the data (Supplementary Figure S2). The concentrated distribution of all QC samples verifies that this method has good stability and reproducibility. PC1 and PC2 represent 14.8% and 11.7% of the total variation in POS, and 13.9% and 11.8% in NEG, respectively. The PCA data showed six clearly separated sample groups, indicating clear separations between the three different treatments of each genotype.

### 3.5. Qualitative and Quantitative Analyses of Metabolites

**Figure 4:**
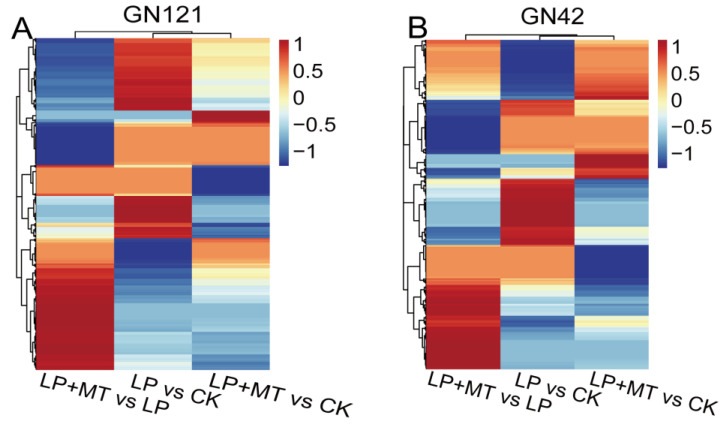
Heat map analysis combined with hierarchical cluster analysis of LP vs. CK, LP+MT vs. CK and LP+MT vs. LP in (**A**) GN121 and (**B**) GN42. Red, increased metabolites; blue, decreased metabolites.

The metabolic changes of 36 barley root samples (2 barley genotypes × 3 treatments (CK, LP, and LP+MT) × 6 biological replications) were studied by UPLC/MS. A total of 1701 known metabolites were annotated through the common metabolite database, of which 1121 were in positive ion (PI) mode and 580 in negative ion (NI) mode (Supplementary Table S2). The OPLS-DA (Supplementary Figure S3) plots revealed that the metabolites of these treatments were well separated. In 12 OPLS-DA plots, the score plots of the total variance and the Q2 value accounted for 37.7% to 55.5%, and 75.6% to 94.2%, respectively. Furthermore, the permutation test of the OPLS-DA model showed that the values of R2 and Q2 in the replacement were lower than those in the original model (Supplementary Figure S4). According to VIP > 1, *p* < 0.05 and fold change >1 or <−1, we identified differential accumulation metabolites (DAMs) in the comparison groups. In this context, 408 and 363 DAMs were identified in GN121 and GN42, respectively, of which 236 were observed in both genotypes (Figure 4; Supplementary Table S2). Most of the DAMs were divided into 47 known categories according to their properties, such as carboxylic acids and derivatives, fatty acyls, organooxygen compounds, benzene and substituted derivatives, steroids and steroid derivatives, indoles and derivatives.

### 3.6. Global Metabolic Changes under LP Stress and Exogenous Melatonin Treatments

**Figure 5:**
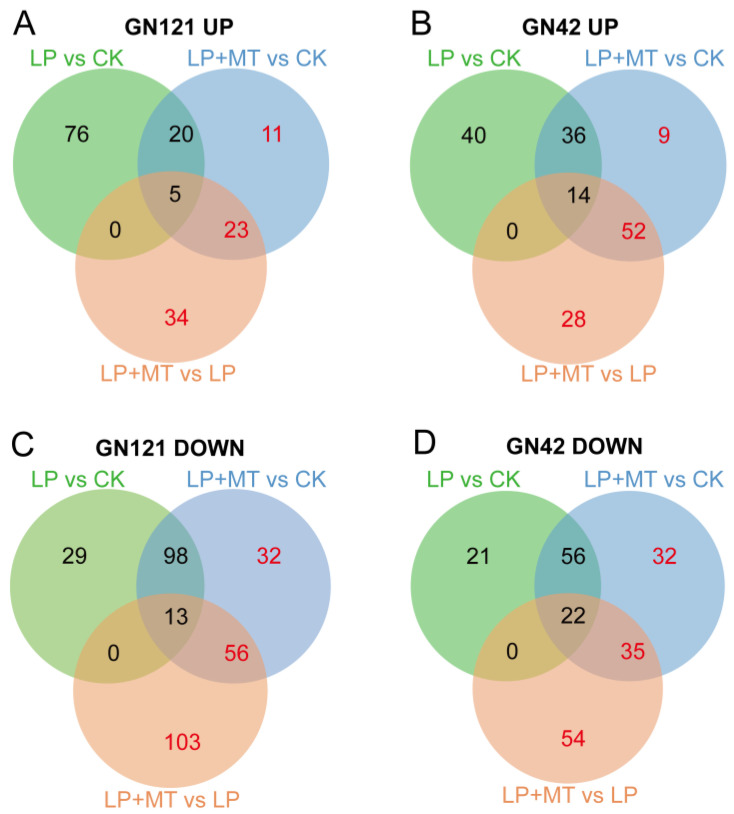
Venn diagram analysis of increased DAMs in (**A**) GN121 and (**B**) GN42, and decreased DAMs in (**C**) GN121 and (**D**) GN42. The red numbers in the Venn diagram indicate the DAMs affected by the “melatonin effect”.

To better understand the metabolic changes due to the exogenous melatonin treatment in the different genotypes of barley exposed to LP stress, we performed a comparative metabolite profiling analysis of GN121 and GN42 barley following the CK, LP and LP+MT treatments (Figure 5). In GN121, the abundance of 101, 59, 62 and 140, 199, 172 metabolites in LP vs. CK, LP+MT vs. CK and LP+MT vs. LP groups increased and decreased, respectively. Similarly, the abundance of 90, 111, 94 and 99, 145, 111 metabolites in LP vs. CK, LP+MT vs. CK and LP+MT vs. LP groups of GN42 increased and decreased, respectively. Furthermore, in order to elucidate the “melatonin effect” in barley roots, we focused on the metabolite contents, except for LP vs. CK groups in the Venn diagram.

There are 68 and 191 metabolites that were increased and decreased, respectively, only in the GN121 roots after exogenous melatonin. Among them, the 68 increased DAMs classification mainly includes carboxylic acids and derivatives, fatty acyls, benzene and substituted derivatives. The 191 decreased DAMs classification mainly includes carboxylic acids and derivatives, fatty acyls, steroids and steroid derivatives, organooxygen compounds. Similarly, there are 89 and 121 metabolites that were increased and decreased, respectively, only in the GN42 roots after exogenous melatonin. Among them, the 89 increased DAMs classification mainly includes indoles and derivatives, carboxylic acids and derivatives, glycerophospholipids. The 121 decreased DAMs classification mainly includes carboxylic acids and derivatives, benzene and substituted derivatives, fatty acyls, organooxygen compounds. Thus, exogenous melatonin treatment led to changes in the metabolome of barley roots under LP stress, and it was different between the two LP-tolerance barley.

### 3.7. Changes in P-Containinglobal Metabolic Changes under LP Stress and Exogenous Melatonin Treatments

We found the existence of phosphate groups in some metabolites of glycerophospholipids, purine nucleotides, pyrimidine and organooxygen compounds (Table 1). A total of 37 P-containing DAMs have been identified, of which 26 were identified in GN121 and 15 were identified in GN42; these DAMs are mainly involved in sugar metabolism, nucleotide metabolism and phospholipid metabolism. In detail, glycerophospholipids are the most abundant P-containing metabolites in the two barley roots, including 9 phosphatidylcholine (PC), 7 phosphatidylethanolamine (PE), 1 lysophosphatidylcholine (LPC), 1 phosphatidylserine (PS), 1 phosphatidylinositol (PI) and 7 other glycerophosphate. In addition, we found four metabolites involved in the sugar metabolism pathway with decreased abundance, including glucose 6-phosphate, mannose 6-phosphate, alpha-d-galactose 1-phosphate and 3-Deoxy-2-keto-6-phosphogluconic acid. There are also six metabolites involved in the nucleotide metabolism pathway with decreased abundance, including adenosine 2′,3′-cyclic monophosphate, cytidine 2′,3′-cyclic phosphate, cytidine 3′-monophosphate, uridine 5′-monophosphate (UMP) and adenosine 3′,5′-cyclic monophosphate in GN121, and guanosine 5′-monophosphate in GN42.

### 3.8. Metabolic Pathway Annotation of DAMs after Exogenous Melatonin Treatment

**Figure 6:**
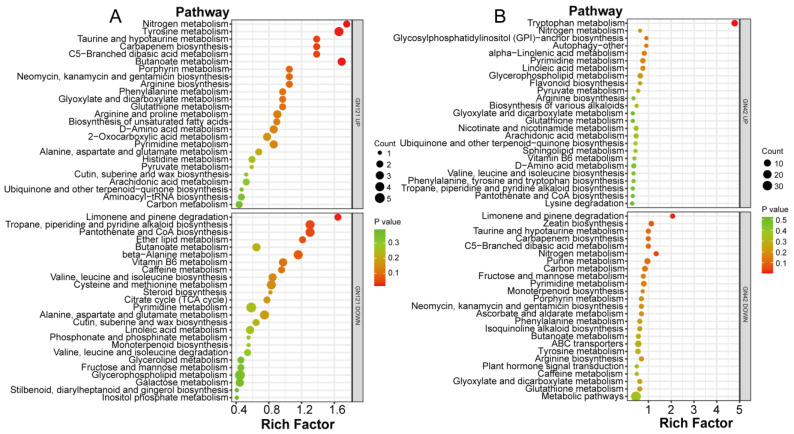
KEGG pathway enrichment analysis of increased and decreased DAMs in barley roots under melatonin treatments. Top 25 KEGG pathways of (**A**) GN121 and (**B**) GN42 are shown.

To identify the main pathways mediated by exogenous melatonin in barley roots in response to LP stress, we mapped differentially abundant metabolites in the GN121 and GN42 roots after the melatonin treatment to KEGG pathways (Figure 6; Supplementary Table S4). In GN121, the metabolite with increased abundance is significantly enriched in six KEGG pathways: nitrogen metabolism (ko00910), butanoate metabolism (ko00650), tyrosine metabolism (ko00350), carbapenem biosynthesis (ko00332), taurine and hypotaurine metabolism (ko00430), and C5-branched dibasic acid metabolism (ko00660), while the metabolite with decreased abundance is significantly enriched in three KEGG pathways: limonene and pinene degradation (ko00903), pantothenate and CoA biosynthesis (ko00770), and tropane, piperidine and pyridine alkaloid biosynthesis (ko00960). In GN42, the metabolite with increased abundance is significantly enriched in the KEGG pathway as tryptophan metabolism (ko00380), while the metabolite with decreased abundance is significantly enriched in three KEGG pathways: limonene and pinene degradation (ko00903), nitrogen metabolism (ko00910), and carbapenem biosynthesis (ko00332).

## 4. Discussion

### 4.1. Exogenous Melatonin and Its Crosstalk with Plant Hormones Regulates Barley Root Growth under LP Stress

**Figure 7:**
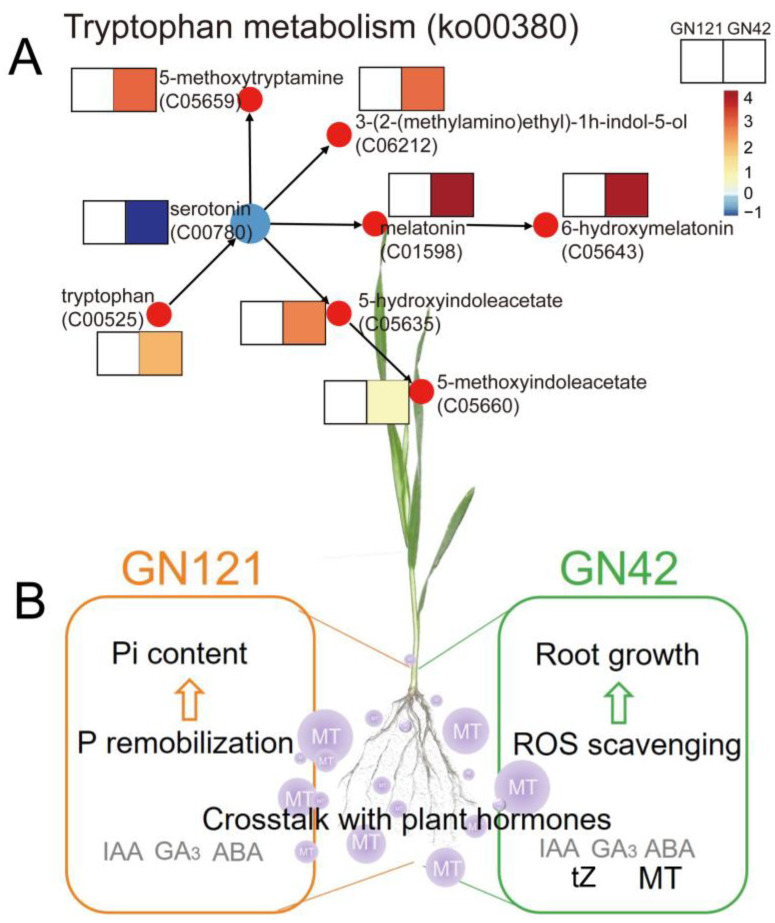
(**A**) Effect of melatonin on DAMs levels related to tryptophan metabolism pathway. (**B**) A summarized model showing the exogenous melatonin effects on two genotypes of barley root under LP stress.

Pi deficiency is a major threat to sustainable crop production globally. In addition to fertilizer application, the application of exogenous growth regulators to cope with plant nutrient deficiency is a good agronomic management method [51]. In plants, melatonin has been extensively reported as an indole hormone and antioxidant to regulate plant growth under abiotic stress. It has been proven that barley is a species with high levels of melatonin and that levels vary from variety to variety [52]. However, the role of exogenous melatonin in the LP stress response of barley with different tolerance is still poorly understood. Our study showed that exogenous melatonin treatment increased endogenous melatonin content in GN42 roots, but had no significant effect on GN121. The content measurement results also showed that, compared with GN42, GN121 was a barley genotype with higher melatonin content, and its endogenous melatonin content increased significantly under LP stress. In addition, exogenous melatonin pretreatment increased total phosphorus content of roots and alleviated the inhibition of LP stress on the growth of barley seedlings, and especially significantly increased root length of GN42 (Figure 1A). Previous studies have shown that the metabolism of melatonin is often more efficient than the synthesis, and the metabolites are also more stable, which is mainly caused by the difference in enzyme activity between the synthesis of melatonin and the metabolic pathway [53]. This indicates that in the two genotypes of barley, a potential relationship exists between LP-tolerance and melatonin metabolism. It is well known that tryptophan is an important substrate for the synthesis of melatonin. In this study, KEGG analysis showed that exogenous melatonin significantly promoted the accumulation of metabolites of tryptophan metabolism (ko00380) pathway in GN42 (Figure 7A). In addition to serotonin, a key precursor of melatonin synthesis, nine other tryptophan metabolites were significantly accumulated under exogenous melatonin treatment. This suggests that exogenous melatonin plays an important role in regulating endogenous melatonin synthesis and metabolism of barley GN42 roots under LP stress. Therefore, we speculated that LP-sensitive barley was also sensitive to exogenous melatonin treatment.

Melatonin has multiple regulatory effects and interacts with other plant growth regulators such as auxin, gibberellin (GA), abscisic acid (ABA), cytokinins, and other regulatory hormones [54,55]. In this study, the hormone determination results showed that exogenous melatonin treatment promoted IAA and GA3 in barley roots, but inhibited ABA synthesis. Previous studies have shown that melatonin regulates root growth in a concentration-dependent manner, analogously to IAA [56]. Chen et al. [57] reported that the exogenous application of 0.1 mM melatonin not only promoted the root growth of wild mustard seedlings, but also increased the endogenous levels of free IAA in roots. In general, a low concentration of melatonin can promote the formation and development of lateral roots, while a high concentration of it inhibits root growth [58], and it is believed that the growth promotion effect of MT may be caused by the increase of IAA content. In addition, melatonin pretreatment affected ABA biosynthesis-related gene expression and upregulated ABA catabolism genes [59,60]. Chen et al. [61] found that melatonin treatment at 20 μM down-regulated GhDPBF2 expression and up-regulated GhABF2 expression in ABA signaling pathway under salt stress. Similarly, it has been shown that melatonin treatment induced up-regulated gene expression of GA receptor proteins GhGID1B and GhGID1C, resulting in increased GA_3_ content in salt-stressed cotton. These results suggested that exogenous melatonin can promote root growth under LP stress by regulating GA_3_ synthesis and ABA decomposition; this result is consistent with our findings. In addition, we found that melatonin treatment reduced the metabolites of zeatin biosynthesis pathway, including s-methyl-5′-thioadenosine (C00170) and isopentenyladenine (C04083) in GN121 and GN42, and trans-zeatin (C00371) in GN42; the hormone determination results also showed that the tZ content of GN42 decreased under MT treatment. tZ is regarded as the most abundant cytokinin type in *Arabidopsis*, and its concentration is reduced by Pi starvation, whereas another zeatin isomer, cis-zeatin (cZ), is significantly increased under Pi starvation conditions [62]. Moreover, it has been shown that maintaining a high cZ/tZ ratio under Pi starvation promotes root elongation, which in turn increases the ability of roots to absorb Pi from the surrounding environment [63]. Recently, a study has also reported that melatonin antagonized the inhibitory effect of cytokinin 6-benzylaminopurine on primary root elongation in *Arabidopsis* [64]. Thus, these results suggested that melatonin may interact with other plant hormones in barley in response to LP stress.

### 4.2. Exogenous Melatonin Alleviated the Oxidative Stress of Barley Roots under LP Stress

In plants, nutrient deficiency generally induces oxidative stress by triggering the production and accumulation of ROS, and excess ROS often leads to cell damage [60]. Previous studies reported that LP stress increased the accumulation of H_2_O_2_ and O^2−^ in tomato plants. Our study found the production of H_2_O_2_ and O^2−^ were significantly increased under LP stress, especially in GN42. In addition, the use of NBT histochemical staining to visualize O^2−^ provided findings of accumulation consistent with the biochemical results (Figure 3). Therefore, the effective removal of ROS is particularly important for plants to deal with LP stress. Melatonin is widely regarded as an antioxidant that plays an important role in scavenging ROS [65,66], and exogenous melatonin has been shown to play an important role in alleviating oxidative stress in crops [29,67,68]. In this study, the application of exogenous melatonin remarkably attenuated the accumulation of ROS in barley roots induced by LP stress; especially the O^2−^ content was significantly reduced. In addition, the activities of antioxidant enzymes, such as SOD, POD and CAT, were markedly increased in melatonin-pretreated roots under LP stress, certifying the function of melatonin in regulating antioxidant enzymes, especially in GN42. In addition, metabolomic analysis showed that the abundance of 4′, 5-dihydroxy-7-methoxyflavanone, a flavonoid metabolite, increased by eightfold in GN42 under melatonin treatment. Consistently, it has been reported that melatonin plays a positive role in the synthesis of flavonoids, which is beneficial to enhance the antioxidant capacity of plants [69]. In contrast, we found that melatonin reduced the metabolic abundance of some non-enzymatic antioxidants, including ascorbate and aldarate metabolism (galactarate and gulonic gamma-lactone), glutathione metabolism (glutamic acid), vitamin A (retinol) in GN42, and galactarate and retinol were reduced in GN121. This is inconsistent with many previous studies reporting that melatonin treatment increased the content of non-enzymatic antioxidants [28,70]. Melatonin is known to be a strong antioxidant, which can directly combine with reactive oxygen radicals and active nitrogen radicals to reduce cell damage, and its metabolites often have antioxidant effects, such as 6-hydroxymelatonin. Therefore, we speculated that exogenous melatonin might have antagonistic effects with non-enzymatic antioxidants in barley roots. Zhang et al. [71] reported that exogenous melatonin enhanced dark-induced ROS scavenging in perennial ryegrass leaves by regulating the SOD-CAT antioxidant pathway, while no significant effect or inhibition was observed of the metabolites of ascorbate-glutathione and glutathione peroxidase pathways, such as free AsA, DHA, GSH and GSSG. This indicates that exogenous melatonin has specific regulation on the antioxidation system.

Because lipids are one of the oxidative targets of ROS overload in plants, lipid peroxidation occurs when plant membrane phospholipids are attacked by ROS, leading to damage to the cell membrane or organelle membranes [72]. Malodialdehyde (MDA) in lipid peroxidation is often a key indicator of lipid membrane damage caused by environmental stress [73]. Previous studies suggested that the MDA content in many plants was significantly increased under LP treatment [74,75]. In this study, we found that MDA content in barley of both genotypes increased significantly under LP stress compared with the control. However, exogenous melatonin treatment only significantly reduced MDA content in GN42. This result suggested that exogenous melatonin played a positive role in alleviating the lipid peroxidation caused by LP stress of in LP-sensitive barley. In addition, several exogenous metabolites involved in the arachidonic acid pathway under exogenous melatonin treatment were identified, including prostaglandin (PG) i2 (21.7-fold increase) and leukotriene (LT) f4 (6.7-fold increase) in GN121, and 6-ketoprostaglandin e1 (1.4-fold decrease) in GN42. Arachidonic acid (AA) produces PGs and LTs through the cyclooxygenase pathway, and PG is a precursor of MDA synthesis [76]. Higher lipid peroxidation levels in GN121 may be related to plasma membrane remodeling; this is discussed in more detail later. We found that melatonin significantly promoted the clearance of phospholipids in GN121 to alleviate phosphorus deficiency in cells, and the large amount of AA produced by phospholipid metabolism may lead to the accumulation of PGs and LTs in GN121. Thus, the regulation of exogenous melatonin on LP stress in barley may involve the complex crosstalk of several metabolic pathways. In conclusion, exogenous melatonin can alleviate LP mediated ROS damage by enhancing the antioxidant capacity of barley roots, which in turn reduces the LP-tolerance gap between GN42 and GN121.

### 4.3. Exogenous Melatonin Promoted the Root P Remobilization in LP-Tolerant Barley

In addition to the use of various strategies to efficiently absorb Pi from the environment, root P remobilization is an important strategy for plants to adapt to phosphorus deficiency [77]. The organic P of plant tissues exists in the form of sugars, nucleic acids, phospholipids, low-molecular-weight P-ester metabolites, and phosphorylated proteins [78]. Under LP conditions, plants can release Pi by scavenging P-containing metabolites to maintain P homeostasis [79]. It has been reported that the concentrations of P-containing sugars, cholines, and nucleotides were decreased in Pi-deficient *Stylosanthes* roots [80]. Such findings have also been reported in *Arabidopsis* [81], barley [82], and white lupin (*Lupinus albus*) [2]. In this study, 92.3% (24/26) of P-containing DAMs in GN121 and 40.0% (6/15) of P-containing DAMs in GN42 were reduced due to melatonin effect, and these reduced metabolites were mainly involved in the metabolism of sugars, nucleotides and phospholipids. For example, the abundances of mannose 6-phosphate and glucose 6-phosphate in GN121 increased significantly under LP stress, while exogenous melatonin dramatically reduced their abundance. Nucleic acid is an important P-containing component in plant cells, and its total phosphorus accounts for about 40–60% of the organic P pool [78]. Therefore, it has been suggested that plants may respond to Pi starvation by inhibiting the synthesis and promoting the degradation of P-containing nucleic acids. For example, RNA content in *Arabidopsis thaliana* shoots decreased dramatically under P starvation compared with that in P-sufficient plants [83]. Müller et al. [2] also reported that Pi limitation led to a decrease in the concentration of nucleic acid in white lupin. In this study, we found that the abundance of five metabolites in GN121 and one metabolite in GN42 involved in the nucleotide metabolism pathway decreased due to melatonin’s effects. Thus, we speculated that melatonin could improve the tolerance of barley to LP stress by enhancing the scavenging of P-containing nucleic acid. Furthermore, membrane lipid remodeling, in which membrane phospholipids are replaced by lipids that do not contain P, is thought to be a major response to Pi starvation in plants [79], and this response may vary between genotypes. Mehra et al. [84] reported that there was lower expression of lipid-remodeling genes in roots of LP-tolerant rice genotypes under Pi deficiency than in LP-sensitive rice genotypes. More recently, a study also showed that the glycerin-3-phosphate content in leaves of wild bald soybean seedlings under LP stress decreased more significantly than that of common wild soybean [85]. We found that exogenous melatonin promoted the degradation of phospholipids. Most phosphocholine (PC), phosphoylethanolamine (PE) and lysophosphatidylcholine (LPC) in GN121 were decreased significantly, while acetylcholine (a precursor of synthetic PC) was significantly increased, suggesting that exogenous melatonin plays a key role in membrane lipid remodeling of LP-tolerant barley. In conclusion, exogenous melatonin enhances P remobilization in LP-tolerant barley and helps to release more Pi in response to LP stress.

To intensively research the metabolic process of LP response regulated by melatonin, we mainly analyzed the differential metabolites involved in root growth and development under melatonin pretreatment conditions and integrated them into a metabolic model (Figure 7B). We found that exogenous melatonin had different metabolic patterns in different LP-tolerant barley, which mainly regulated the endogenous plant hormones and anti-oxidative stress in GN42 to cope with LP damage, and promoted the P remobilization in GN121 to supplement the lack of Pi in roots. Therefore, we believe that melatonin plays a broad role in response of barley to LP stress. However, plant responses to changes in Pi involve complex patterns between multiple biological processes. For example, how exogenous melatonin regulates the balance between its antioxidant activity and cellular antioxidant system, and the possible relationship between cellular lipid peroxidation and membrane lipid remodeling, are all of great significance for studying the function of melatonin in plants.

## 5. Conclusions

This is the first study demonstrating the effects of exogenous melatonin in alleviating LP stress in crops with different genotypes. Here, we show that exogenous melatonin could promote the growth of barley roots under LP stress, especially by increasing the primary root length. Untargeted metabolomic analysis revealed that exogenous melatonin treatment could enhance barley root tolerance to LP stress by regulating endogenous hormone metabolism, antioxidant system and promoting P remobilization processes, which are mainly achieved through the role of melatonin as a plant hormone and antioxidant. Although there were differences in the metabolism of melatonin to LP stress in different barley genotypes, appropriate treatment was beneficial in reducing the genetic gap of LP tolerance. This study provides useful information on the differential metabolic response of barley roots to LP stress in different genotypes and the protective role of melatonin in enhancing LP tolerance, which should facilitate future Pi-efficient crop breeding.

## Figures and Tables

**Table 1:** The P-containing DAMs in GN121 and GN42 after exogenous melatonin treatment.

Genotype	Index	Log2_FC *	MS2_Name
GN121	M403T35_NEG	0.8825	1-o-(9z-octadecenyl)-sn-glycero-2,3-cyclic-phosphate
	M811T39_POS	0.3398	1-octadecanoyl-2-octadecenoyl-sn-glycero-3-phosphocholine
	M715T38_1_NEG	−0.4421	2-linoleoyl-1-palmitoyl-sn-glycero-3-phosphoethanolamine
	M431T245_NEG	−0.6881	1-Palmitoyl Lysophosphatidic Acid
	M618T36_NEG	−0.719	N-palmitoyl-d-erythro-dihydroceramide-1-phosphate
	M752T151_2_NEG	−0.7461	Pe (16:0/8-hepe)
	M757T146_1_POS	−0.9683	PC (16:0/16:0)
	M860T193_NEG	−1.0082	Pi 36:3
	M409T252_NEG	−1.1	1-hexadecanoyl-2-sn-glycero-3-phosphate
	M509T145_NEG	−1.1835	1-oleoyl-2-hydroxy-sn-glycero-3-phospho-(1′-rac-glycerol)
	M241T395_NEG	−1.2173	alpha.-d-galactose 1-phosphate
	M322T432_NEG	−1.2436	Cytidine 3′-monophosphate
	M850T198_POS	−1.2817	1,2-dipalmitoyl-sn-glycero-3-phospho-(1′-myo-inositol)
	M774T71_NEG	−1.3111	1,2-distearoyl-sn-glycero-3-phospho-l-serine
	M328T265_NEG	−1.3342	Adenosine 3′,5′-cyclic monophosphate
	M323T412_1_NEG	−1.4557	Uridine 5′-monophosphate (UMP)
	M304T319_NEG	−1.482	Cytidine 2′,3′-cyclic phosphate
	M428T93_POS	−1.5782	Arachidonoyl ethanolamide phosphate
	M330T266_POS	−1.6632	Adenosine 2′,3′-cyclic monophosphate
	M520T186_2_POS	−1.9729	Lpc 18:2
	M184T486_POS	−2.1167	1-o-hexadecyl-2-deoxy-2-thio-s-acetyl-sn-glyceryl-3-phosphorylcholine
	M214T390_NEG	−2.2073	sn-Glycerol 3-phosphoethanolamine
	M258T384_POS	−2.3738	Glycerophosphocholine
	M171T430_NEG	−2.4824	Glycerophosphate (2)
	M243T470_POS	−3.0013	glucose 6-phosphate
	M259T468_NEG	−3.9351	mannose 6-phosphate
GN42	M743T183_NEG	2.6019	1-stearoyl-2-linoleoyl-sn-glycero-3-phosphoethanolamine
	M753T183_POS	1.9118	1,2-dipalmitoleoyl-sn-glycero-3-phosphocholine
	M767T183_POS	1.8359	1,2-dioleoyl-sn-glycero-3-phosphoethanolamine
	M821T200_1_NEG	1.4875	1-stearoyl-2-linoleoyl-sn-glycero-3-phosphocholine
	M508T186_POS	1.2428	1-(1z-octadecenyl)-sn-glycero-3-phosphocholine
	M520T186_2_POS	1.0943	Lpc 18:2
	M835T39_1_POS	0.3304	1-stearoyl-2-docosahexaenoyl-sn-glycero-3-phosphocholine
	M452T200_NEG	0.3301	1-palmitoyl-2-hydroxy-sn-glycero-3-phosphoethanolamine
	M811T39_POS	0.2905	1-octadecanoyl-2-octadecenoyl-sn-glycero-3-phosphocholine
	M279T109_NEG	−0.3926	3-Deoxy-2-keto-6-phosphogluconic acid
	M774T71_NEG	−0.4553	1,2-distearoyl-sn-glycero-3-phospho-l-serine
	M715T38_1_NEG	−0.5803	2-linoleoyl-1-palmitoyl-sn-glycero-3-phosphoethanolamine
	M841T144_NEG	−0.767	Pc (16:0e/8-hepe)
	M817T146_NEG	−1.0013	Pc (16:1e/9-hode)
	M362T437_NEG	−1.5738	Guanosine 5′-monophosphate

* The log2_FC value of the intersection of LP+MT vs. CK and LP+MT vs. LP is the average of the two comparison groups.

## Data Availability

The datasets used and/or analyzed during the current study are available from the corresponding author on reasonable request.

## Supplementary Materials

The following supporting information can be downloaded at: https://www.mdpi.com/article/10.3390/cells12101397/s1, Figure S1: The chromatogram of nine representative metabolites. (A) 6-hydroxymelatonin, (B) serotonin, (C) trans-zeatin, (D) glucose 6-phosphate, (E) Pc (16:0e/8-hepe), (F) Lpc 18:2, (G) 6-ketoprostaglandin e1, (H) prostaglandin i2, (I) leukotriene f4; Figure S2: Overall score plots of the PCA model with quality control in (A) positive and (B) negative ion modes; Figure S3: OPLS-DA scores of GN121 in (A) LP vs. CK, (B) LP+MT vs. CK, (C) LP+MT vs. LP and GN42 in (D) LP vs. CK, (E) LP+MT vs. CK, (F) LP+MT vs. LP in positive ion mode; OPLS-DA scores of GN121 in (G) LP vs. CK, (H) LP+MT vs. CK, (I) LP+MT vs. LP and GN42 in (J) LP vs. CK, (K) LP+MT vs. CK, (L) LP+MT vs. LP in negative ion mode; 121, LP-tolerant barley (GN121); 42, LP-sensitive barley (GN42); CK, normal Pi (0.397 mM); LP, low Pi (0.0397 mM); LP+MT, low Pi (0.0397 mM) + 30 μM melatonin; Figure S4: Permutation tests of the OPLS-DA model of GN121 in (A) LP vs. CK, (B) LP+MT vs. CK, (C) LP+MT vs. LP and GN42 in (D) LP vs. CK, (E) LP+MT vs. CK, (F) LP+MT vs. LP in positive ion mode; Permutation tests of the OPLS-DA model of GN121 in (G) LP vs. CK, (H) LP+MT vs. CK, (I) LP+MT vs. LP and GN42 in (J) LP vs. CK, (K) LP+MT vs. CK, (L) LP+MT vs. LP in negative ion mode; Table S1: The identified metabolites of barley roots in positive ion (PI) mode and in negative ion (NI) mode; Table S2: The known metabolites identified in GN121 and GN42; Table S3: The DAMs of GN121 and GN42 in LP vs. CK, LP+MT vs. CK, LP+MT vs. LP groups; Table S4: KEGG pathway enrichment analysis of DAMs in GN121 and GN42 after exogenous melatonin treatment.
