# Supplementary material for: Exogenous Melatonin Enhances the Low Phosphorus Tolerance of Barley Roots of Different Genotypes
Source: Cells. 2023 May 16;12(10):1397. doi: 10.3390/cells12101397 (PMC10217165; doi:10.3390/cells12101397)
Supplement: Supplementary file 1 [file cells-12-01397-s001.zip › cells-2312473-supplementary.pdf]

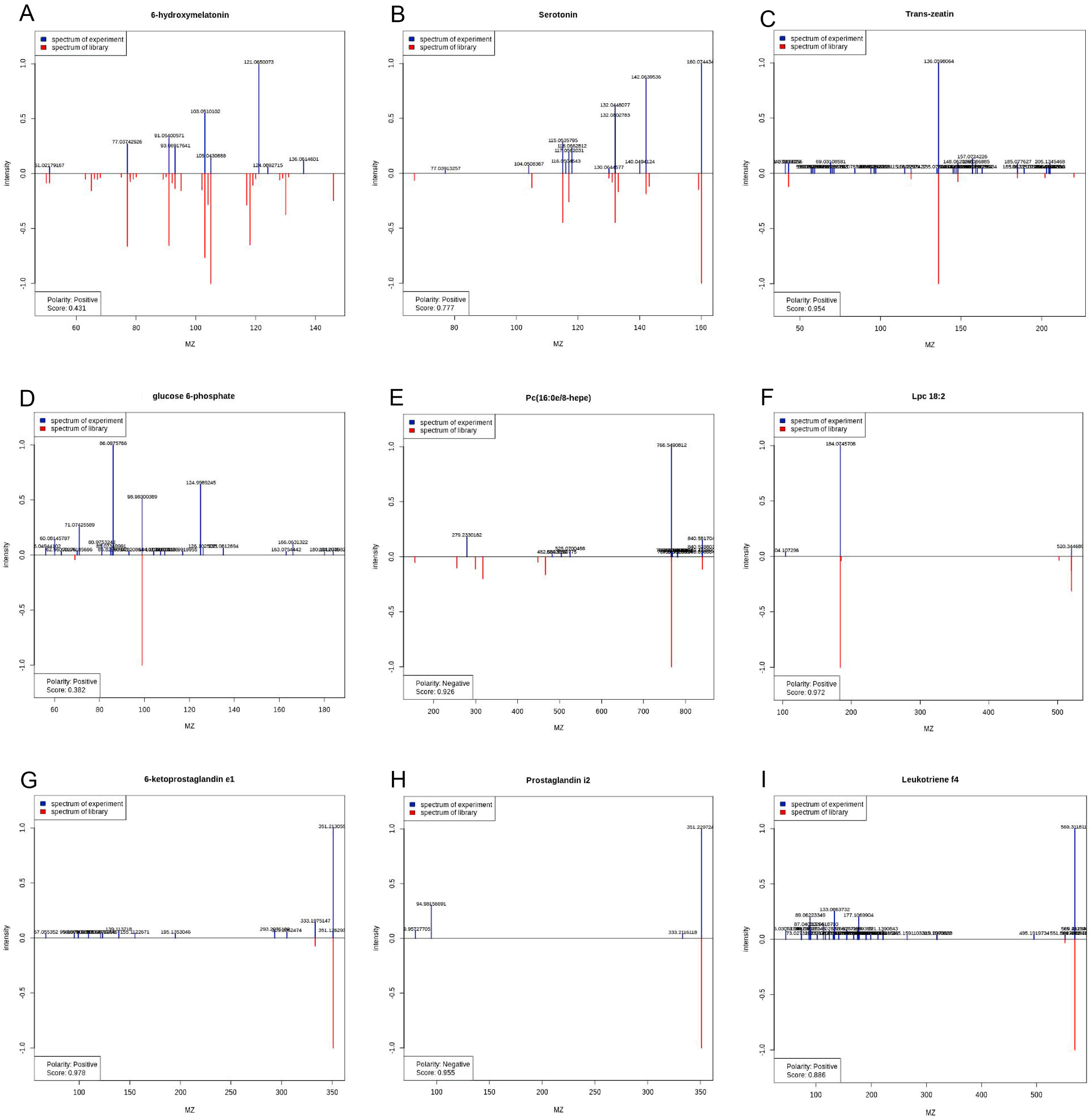

Figure S1. The chromatogram of nine representative metabolites. (**A**) 6-hydroxymelatonin, (**B**) serotonin, (**C**) trans-zeatin, (**D**) glucose 6-phosphate, (**E**) Pc (16:0e/8-hepe), (**F**) Lpc 18:2, (**G**) 6-ketoprostaglandin e1, (**H**) prostaglandin i2, (**I**) leukotriene f4.

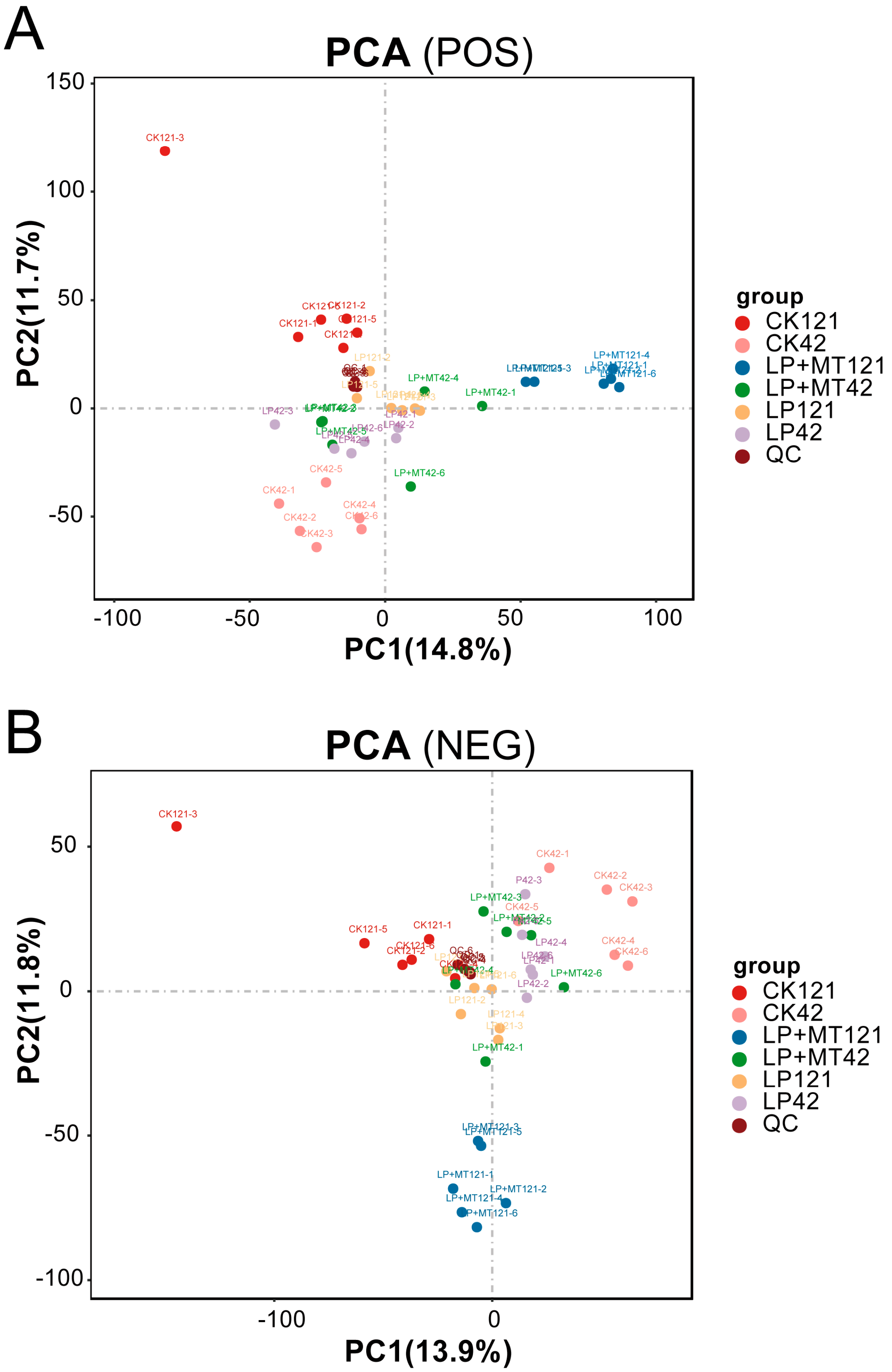

Figure S2. Overall score plots of the PCA model with quality control in (**A**) positive and (**B**) negative ion modes.

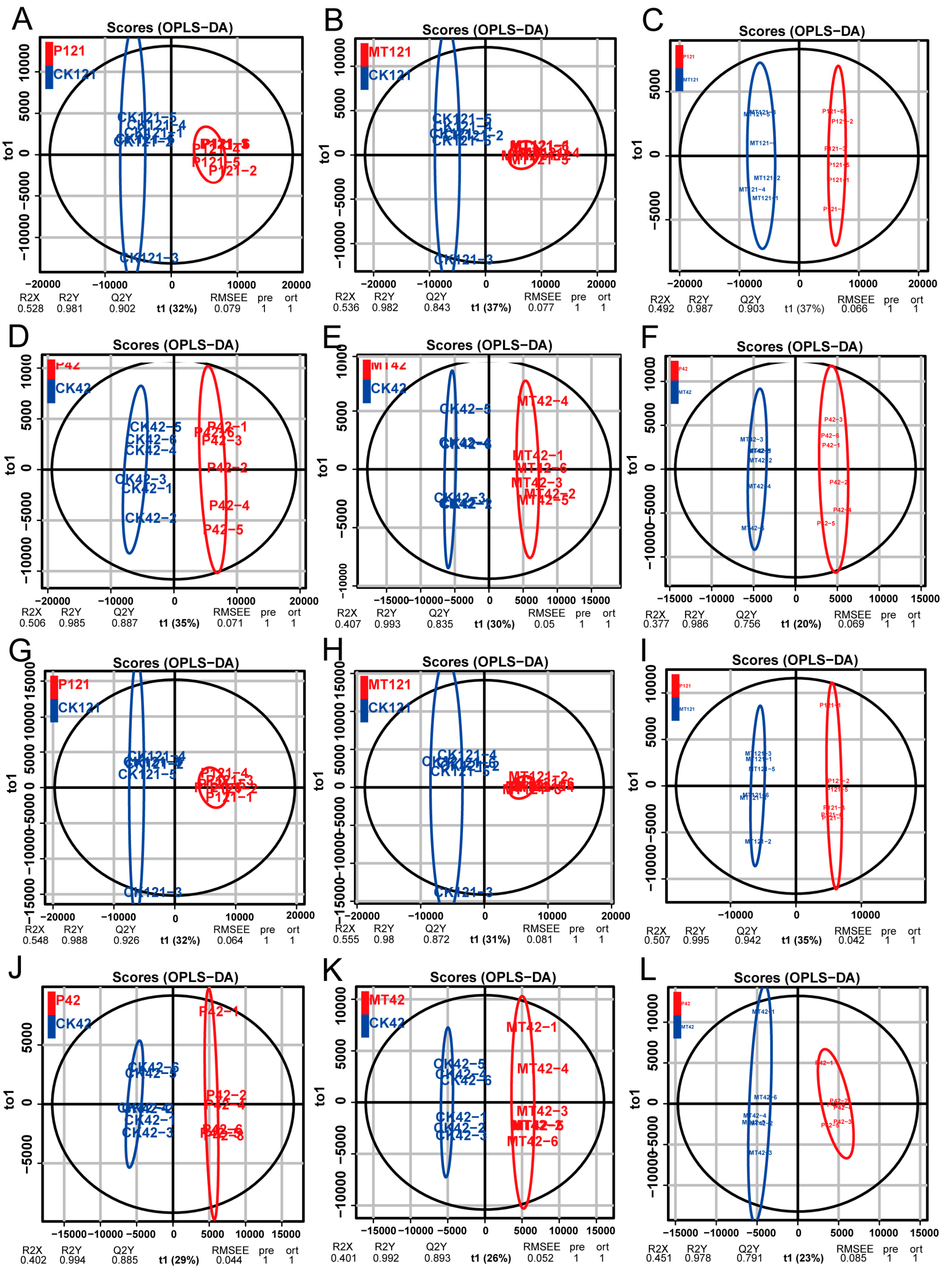

Figure S3. OPLS-DA scores of GN121 in (**A**) LP vs. CK, (**B**) LP+MT vs. CK, (**C**) LP+MT vs. LP and GN42 in (**D**) LP vs. CK, (**E**) LP+MT vs. CK, (**F**) LP+MT vs. LP in positive ion mode; OPLS-DA scores of GN121 in (**G**) LP vs. CK, (**H**) LP+MT vs. CK, (**I**) LP+MT vs. LP and GN42 in (**J**) LP vs. CK, (**K**) LP+MT vs. CK, (**L**) LP+MT vs. LP in negative ion mode; 121, LP-tolerant barley (GN121); 42, LP-sensitive barley (GN42); CK, normal Pi (0.397 mM); LP, low Pi (0.0397 mM); LP+MT, low Pi (0.0397 mM) + 30 μM melatonin.

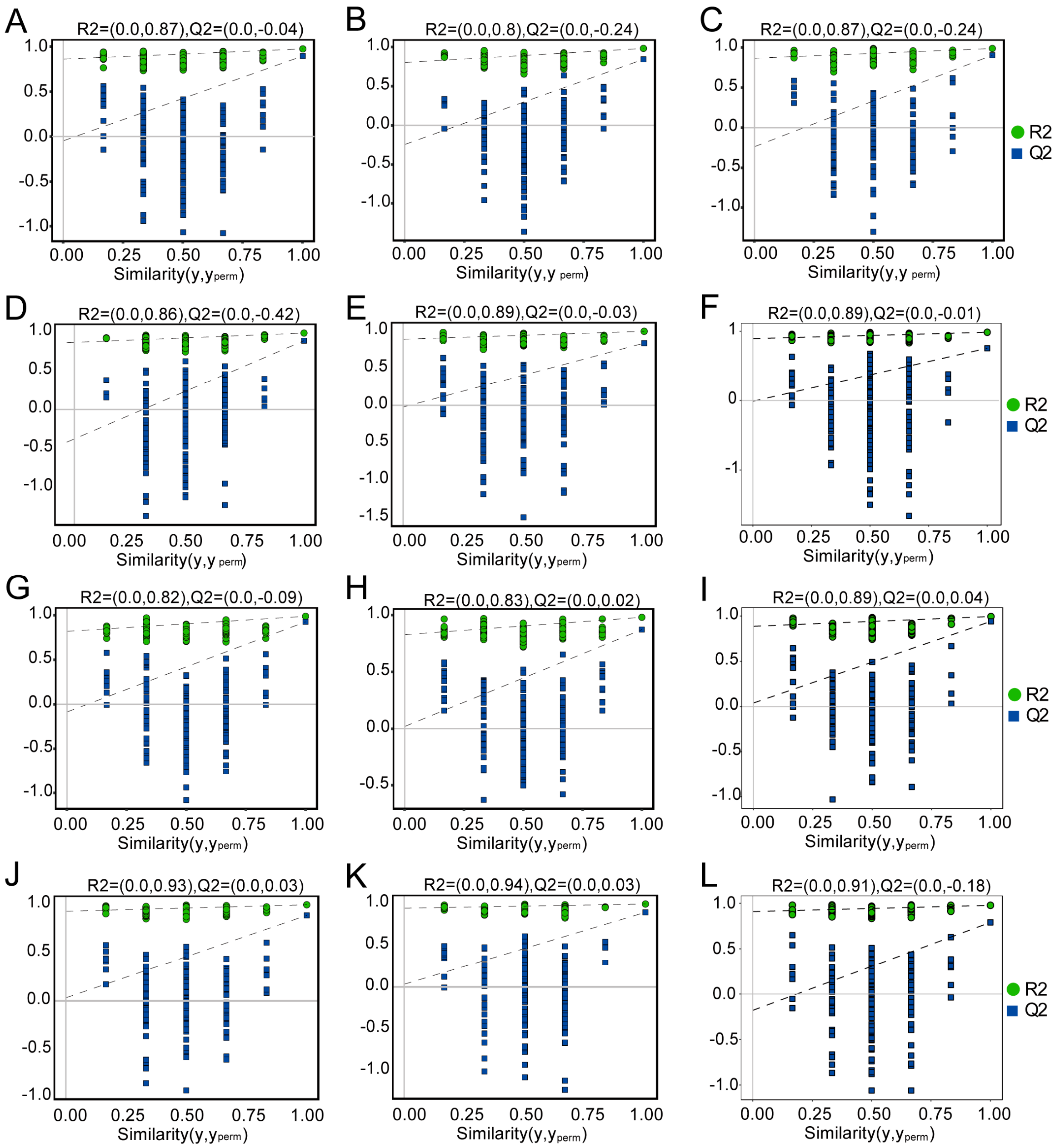

Figure S4. Permutation tests of the OPLS-DA model of GN121 in (**A**) LP vs. CK, (**B**) LP+MT vs. CK, (**C**) LP+MT vs. LP and GN42 in (**D**) LP vs. CK, (**E**) LP+MT vs. CK, (**F**) LP+MT vs. LP in positive ion mode; Permutation tests of the OPLS-DA model of GN121 in (**G**) LP vs. CK, (**H**) LP+MT vs. CK, (**I**) LP+MT vs. LP and GN42 in (**J**) LP vs. CK, (**K**) LP+MT vs. CK, (**L**) LP+MT vs. LP in negative ion mode.
